# Effects of SGLT2I Therapy on Tubular Reabsorption and Tubular Epithelial Stress Injury in Patients With CKD

**DOI:** 10.1016/j.xkme.2026.101416

**Published:** 2026-05-26

**Authors:** Ann-Kathrin C. Schäfer, Dennis Pieper, Bilgin Bayram, Jamil Ajrab, Fani Delistefani, Michael Zeisberg, Michael J. Koziolek, Manuel Wallbach

**Affiliations:** 1 Department of Nephrology and Rheumatology, University Medical Center Göttingen, Göttingen, Germany; 2 German Center for Cardiovascular Research, Partner Site Göttingen, Germany

**Keywords:** α-1-microglobulin, chronic kidney disease, Dickkopf-3, sodium-glucose cotransporter-2 inhibitor, tubular proteinuria

## Abstract

**Rationale & Objective:**

Sodium-glucose cotransporter-2 inhibitors (SGLT2I) are a standard of care treatment for chronic kidney disease (CKD). However, the effects of SGLT2I on tubular function and stress are rare.

**Study Design:**

A monocentric, prospective, observational study.

**Setting & Participants:**

Patients with CKD who were referred to the outpatient clinic of the Department of Nephrology at Göttingen University Hospital and for whom SGLT2I therapy was indicated as part of standard treatment were prospectively included and treated with SGLT2I in accordance with current guidelines.

**Analytical Approach:**

Urine samples were collected, and the levels of urinary α1-microglobulin (uα-1-MG), urinary Dickkopf-3 (uDKK3), and urinary albumin creatinine ratio (UACR), each normalized to urinary creatinine, were measured at baseline and after 6 months of therapy.

**Results:**

Total of 57 patients were included. The mean age was 66.4 ± 12.4 years; 42.1% were female. At baseline, mean estimated glomerular filtration rate was 42.0 ± 15.3 mL/min/1.73m^2^. Within 6 months, the mean estimated glomerular filtration rate decreased by −2.1 ± 7.6 mL/min/1.73 m^2^ (*P* = 0.04). While overall patients, UACR (*P* = 0.23) and uDKK3 (*P* = 0.87) remained unchanged, there was a significant increase in median uα-1-MG within 6-months (+9.7 mg/g creatinine [IQR 1.0-22.2]; *P* < 0.001). Although patients with A3 albuminuria (n = 14) showed only a numerical increase of median uα-1-MG level (+4.6 mg/g creatinine [IQR −7.1 to 20.8]; *P* = 0.30), there was a significant increase of median uα-1-MG in stage A1 (n=22, +11.5 mg/g creatinine [IQR 3.8-23.2]; *P* < 0.001) and A2 patients (n = 21, +7.2 mg/g creatinine [IQR 0.7-30.8]; *P* = 0.006).

**Limitations:**

The small sample size, lack of a control group, and the short follow-up duration may restrict the findings.

**Conclusions:**

This study demonstrates an increase in uα-1-MG during SGLT2I treatment in patients with CKD, particularly in UACR stage A1, in which reduction of hyperfiltration is less pronounced. Because uDKK3 as a parameter of tubular damage remained stable, the increase in uα-1-MG may reflect a functional reduction of protein reabsorption and thus reduced intratubular protein overload as a potential nephroprotective effect.

## Plain-Language Summary

This study analyzed urine samples from patients receiving sodium-glucose cotransporter inhibitors (SGLT2I) as part of their treatment for chronic kidney disease. The urinary concentrations of the proteins α-1-microglobulin, Dickkopf-3, and albumin were measured before the start of treatment and after 6 months of therapy. Although the concentrations of albumin and Dickkopf-3 remained unchanged, the urinary concentration of α-1-microglobulin increased significantly during the 6-month SGLT2-I therapy. Because Dickkopf-3 levels as a marker of injury remained constant, the increase in α-1-microglobulin in urine could be an expression of functionally reduced protein reabsorption from the urine and thus reduced protein overload in the kidney cells, possibly representing a kidney-protective effect of SGLT2-I.

Sodium-glucose cotransporter-2 inhibitors (SGLT2I) have emerged as a promising therapeutic class for chronic kidney disease (CKD), demonstrating benefits for both the kidneys and the cardiovascular system, although the exact mechanisms are not yet fully understood.^1^^,^^2^ The nephroprotective effects of SGLT2I are attributed to multiple mechanisms, including their impact on tubuloglomerular feedback, reduction in intraglomerular pressure, and alteration in renal tubular protein handling.^3^, ^4^, ^5^, ^6^ Given that albuminuria and urinary tubular proteins are key markers of kidney damage, it is important to understand their underlying pathophysiology. Albuminuria can result from increased glomerular filtration (glomerular albuminuria) or impaired tubular reabsorption (tubular albuminuria). When the filtered albumin load exceeds the proximal tubule’s limited endocytic capacity, reabsorption becomes insufficient. Under physiological conditions, ∼0.1% of plasma albumin (∼45 g/L) is filtered by the glomerulus. At a glomerular filtration rate (GFR) of ∼180 L/day, this results in a filtered load of ∼8,100 mg/day, with almost all of the filtered albumin being reabsorbed via megalin-mediated and cubilin-mediated endocytosis.^7^, ^8^, ^9^ Tubular dysfunction can be assessed using both functional and structural biomarkers. Functional tubular biomarkers include low molecular weight plasma proteins (LMWP) that are freely filtered and normally reabsorbed in the proximal tubules, whereas an increased urinary excretion indicates impaired reabsorption. Moreover, an alternative explanation involves competitive reabsorption of proteins in the proximal tubule. Experimental and clinical data suggest that albumin and LMWPs share common uptake pathways, and this competition may contribute to increased LMWP excretion in CKD.^10^^,^^11^ The α-1-microglobulin (α-1-MG), a LMWP (∼27 kDa) freely filtered at the glomerulus and reabsorbed by megalin-asscociated endocytosis in the proximal tubule, serves as a sensitive marker for tubular dysfunction.^12^, ^13^, ^14^, ^15^ Its urinary excretion increases with impaired tubular function or reduced reabsorptive capacity and reduced estimated (eGFR).^12^ Structural tubular biomarkers, by contrast, are urinary proteins released directly from damaged or regenerating tubular cells, reflecting structural injury rather than altered reabsorption.^11^ The Dickkopf-3 (DKK3), a 38-kDa glycoprotein secreted under tubular epithelial stress, has emerged as a structural biomarker for tubular injury and fibrosis through activation of the Wnt/β-catenin pathway. Urinary DKK3 (uDKK3) levels correlate with tubular stress and predict short-term decline in eGFR.^16^ Combining functional (eg, α-1-MG) and structural (eg, DKK3) urinary biomarkers allows for a comprehensive assessment of tubular health in CKD. Although SGLT2I have consistently shown reductions in albuminuria, their effects on specific tubular biomarkers such as α-1-MG and DKK3 remain less well understood. The differential impact of SGLT2I on glomerular versus tubular protein handling, may vary with the degree of proteinuria and CKD stage. To assess the effect of SGLT2I treatment, this study evaluates the effect of a 6-month SGLT2I treatment on urinary (uα-1-MG), uDKK3, and urinary albumin creatinine ratio (UACR). By examining both functional and structural parameters of tubular integrity, this work aims to elucidate the mechanisms underlying SGLT2I-mediated changes in tubular proteinuria and its potential role in SGLT2I-mediated nephroprotection.

## Material and Methods

### Study Design and Participants

This is a nonrandomized, monocentric, prospective, observational study. Patients with CKD who were referred to the outpatient clinic of the Department of Nephrology at Göttingen University Medical Center for nephrological consultation from January 2022 to March 2023 and who had an indication for starting novel SGLT2I therapy as part of standard treatment were prospectively included in the study. Patients with pregnancy, breastfeeding, after kidney transplantation or requiring dialysis, with CKD stage >G4, autosomal dominant polycystic kidney disease, current acute kidney injury, postrenal obstruction, pulmonary hypertension due to causes other than heart failure (HF), diabetes mellitus type 1, active malignant disease, or inflammatory or autoimmune disease affecting kidney function; or patients with known nonadherence were excluded. As per approval, dapagliflozin was started up to an eGFR of 25 mL/min/1.73m^2^ and empagliflozin up to an eGFR of 20 mL/min/1.73m^2^. Study visits were performed one day before the start of SGLT2I therapy (dapa- or empagliflozin, each 10 mg/day) and 6 months ± 14 days thereafter. The study complies with the principles of the Declaration of Helsinki and the local ethical committee approved the study protocol (ethical vote number 38/6/21). All patients provided written informed consent. The study was prospectively registered at the German Clinical Trials Register (DRKS00026098).

### Laboratory Measurements

Serum and urine creatinine, uα-1-MG, and urinary albumin were measured as part of routine measurements. Values below the detection limit were assumed to be the lower detection limit.

The eGFR was calculated based on serum creatinine using the CKD-Epidemiology Collaboration equation (CKD-EPI). Serum and urine creatinine were measured using an enzymatic test (MULTIGENT Creatinine (Enzymatic) Kit, Sentinel CH. SpA). The quantification limit for urine creatinine in this assay is 2.2 mg/dL. The determination of urine creatinine is linear from 2.5 to 400 mg/dL.

The coefficient of variation (CV) is ≤ 3.6% for values > 60 mg/dL and the standard deviation is ≤ 3.6 mg/dL for values ≤ 60 mg/dL.

Albuminuria was quantified using a turbidimetric immunoassay (MULTIGENT Microalbumin Assay, Wako Pure Chemical Industries, Ltd). The assay measurement range is between 5.0 and 500.0 μg/mL. The detection limit is 1 μg/mL. The CV is < 5%.

The uα-1-MG was measured using a turbidimetric immunoassay (A1M Architect Diagam). The measurement range of the assay is between 1.29 and 166.6 mg/L. The detection limit is 0.46 mg/L. The CV depends on the uα-1-MG concentration and ranges between 0.5 and 4.0%.

The uDKK3 was measured using a commercial ELISA kit (ReFiNE Dkk3 ELISA, DiaRen UG), which is validated for urine.^17^^,^^18^ Before analysis, the urine samples were clarified by centrifugation for 10 minutes by 2,000 rpm. The residue was diluted 1:10. The test reliably records DKK3 down to 10 pg/mL sample buffer. The intra-assay and inter-assay variability was specified as 1.6% to 2.5%. All urinary biomarkers were normalized to urinary creatinine to account for dilution of the urine.

### Statistical Analysis

The data were analyzed using Graphpad Prism Version 10 (GraphPad Software). The presence of a normal distribution was tested using the D'Agostino & Pearson test. The 2-tailed paired t test or the Wilcoxon signed rank test were used to assess the time-dependent changes in the variables studied. Spearman r was calculated for the correlation analysis. Data are presented as mean ± standard deviation (SD), median, and interquartile range (IQR), or number (n) and percent (%). The significance level was set to α = 5% for all statistical tests.

Because of their clinical relevance and prior literature, demographic and clinical covariates potentially influencing urinary biomarkers were analyzed. These included age, sex, body mass index (BMI), kidney function assessed by eGFR, and comorbidities such as type 2 diabetes mellitus and HF. All covariates were collected at baseline from medical histories and electronic health records. BMI was calculated from patient-reported weight and height. The eGFR was calculated using the CKD-EPI equation based on baseline serum creatinine. Type 2 diabetes mellitus was defined by documented diagnosis. The HF was classified based on baseline echocardiography and concomitant NT-proBNP levels. Associations between covariates and changes in urinary biomarkers were analyzed using multiple linear regression with ordinary least squares. Changes in uDKK3, uα-1-MG, and UACR were calculated as the difference between the baseline and the 6-month values of SGLT2I therapy, which were log10-transformed to approximate a normal distribution.

In addition to age, sex, BMI, eGFR, diabetes status, and HF, baseline levels of the respective urinary biomarkers were included as independent variables. Continuous variables (age, BMI, and eGFR) were mean-centered to facilitate interpretation of regression coefficients.

## Results

### Baseline Characteristics

A total of 57 patients with CKD were included in the study, of those 22 patients showed albuminuria less than 30 mg/g creatinine (stage A1). About 21 patients had albuminuria between 30-300 mg/g creatinine (A2) and 14 patients showed more than 300 mg albumin/g creatinine in urine (stage A3). The complete cohort comprised 24 females (42.1%) and had a mean age of 66.4 ± 12.4 years. The mean BMI was 28.9 ± 5.3 kg/m^2^. Regarding comorbidities, 96.5% had arterial hypertension, 73.7% had hyperlipoproteinemia, and 69.6% had a history of smoking. Diabetes mellitus type 2 was present in 23 patients (40.4%). Cardiovascular diseases were prevalent, with 40.4% of patients diagnosed with coronary artery disease and 59.6% suffering from HF; of those, 14 patients had heart failure with reduced ejection fraction. Peripheral artery disease and cerebrovascular disease were present in 8 (14.0%) and 9 (15.8%) patients, respectively. The treatment of diseases for which the use of SGLT2Is was justified was carried out in accordance with established guidelines. In particular, renin-angiotensin-aldosterone system inhibitors were used extensively (Table 1).

**Table 1:** Baseline Characteristics and Cardiovascular Medication

Parameter	All (N = 57)	A1 (n = 22)	A2 (n = 21)	A3 (n = 14)
**Age, y**	66.4 ± 12.4	67.4 ± 7.8	70.4 ± 12.0	58.6 ± 15.8
**Female sex, n (%)**	24 (42.1%)	13 (59.1%)	6 (28.6%)	5 (35.7%)
**Body mass index, kg/m^2^**	28.9 ± 5.3	28.9 ± 5.3	28.6 ± 5.5	29.5 ± 5.4
**Arterial hypertension, n (%)**	55 (96.5%)	21 (95.5%)	20 (95.2%)	14 (100%)
**Hyperlipoproteinemia, n (%)**	42 (73.7%)	14 (63.6%)	17 (81.0%)	11 (78.6%)
**History of smoking, n (%)**	39 (69.6%)	14 (63.6%)	16 (76.2%)	9 (64.3%)
**Diabetes mellitus type 2, n (%)**	23 (40.4%)	5 (22.7%)	11 (52.4%)	7 (50.0%)
**Atrial fibrillation, n (%)**	18 (31.6%)	8 (36.4%)	8 (38.1%)	2 (14.3%)
**Coronary artery disease, n (%)**	23 (40.4%)	6 (27.3%)	12 (57.1%)	5 (35.7%)
**HF, n (%)**	34 (59.6%)	13 (59.1%)	12 (57.1%)	9 (64.3%)
HFpEF, n (%)	20 (35.1%)	9 (40.1%)	5 (23.8%)	6 (42.9%)
HFrEF, n (%)	14 (24.6%)	4 (18.2%)	7 (33.3%)	3 (21.4%)
**Peripheral artery disease, n (%)**	8 (14.0%)	1 (4.5%)	5 (23.8%)	2 (14.3%)
**Cerebrovascular disease, n (%)**	9 (15.8%)	0 (0.0%)	7 (33.3%)	2 (14.3%)
**COPD, n (%)**	9 (15.8%)	3 (13.6%)	5 (23.8%)	1 (7.1%)
**Medication**				
ACEi	18 (31.6%)	6 (27.3%)	7 (33.3%)	5 (35.7%)
ARB	25 (43.9%)	10 (45.5%)	9 (42.9%)	6 (42.9%)
ARNI	10 (17.5%)	5 (22.7%)	4 (19.0%)	1 (7.1%)
MRA	12 (21.1%)	5 (22.7%)	4 (19.0%)	3 (21.4%)
Diuretics	42 (73.7%)	16 (72.7%)	18 (85.7%)	8 (57.1%)
β-Blocker	45 (78.9%)	18 (81.8%)	17 (81.0%)	10 (71.4%)
CCB	28 (49.1%)	7 (31.8%)	13 (61.9%)	8 (57.1%)
GLP1-RA	5 (8.8%)	1 (4.5%)	2 (9.5%)	2 (14.3%)

*Note:* Baseline characteristics of all patients and patients with A1/A2/A3 proteinuria. Values are mean ± SD or n (%).

Abbreviations: ACEi, ACE inhibitor); ARB, angiotensin receptor blocker; ARNI, angiotensin receptor neprilysin inhibitor; BMI, body mass index; CCB, calcium channel blocker; COPD, chronic obstructive pulmonary disease; GLP1-RA, GLP1 receptor agonist; HF, heart failure; HFpEF, heart failure with preserved ejection fraction; HFrEF, heart failure with reduced ejection fraction; MRA, mineralocorticoid receptor antagonist.

### Course of eGFR within 6 months of SGLT2I therapy

**Figure 1:**
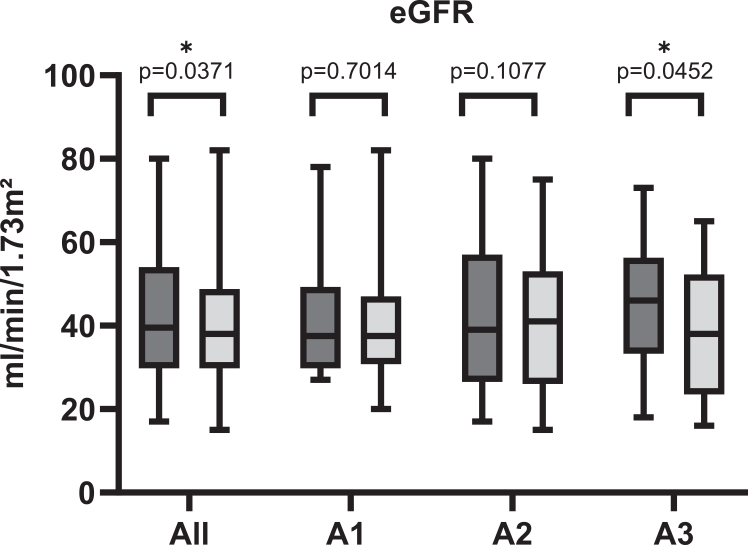
Course of estimated glomerular filtration rate under 6 months of sodium-glucose cotransporter-2 inhibitors therapy in patients with different urinary albumin creatinine ratio stages.

Within 6 months of SGLT2I therapy, the mean eGFR decreased in all patients from 42.0 ± 15.3 to 39.9 ± 15.1 mL/min/1.73m^2^ (*P* = 0.04, Table 2). Regarding patients in the different albuminuria stages, there was no change in the eGFR in patients with albuminuria A1 (*P* = 0.70) and A2 (*P* = 0.11), whereas there was a reduction of mean eGFR from 44.1 ± 16.6 to 39.2 ± 15.9 mL/min/1.73m^2^ in patients with stage A3 (*P* = 0.05; Fig 1).

**Table 2:** Course of Renal Markers in the Different UACR Stages During 6 Months of SGLT2I Therapy

Parameter	Group	Baseline	Month 6	*P*
**eGFR (mL/min/1.73 m^2^)**	All	42.0 ± 15.3	39.9 ± 15.1	0.04^a^
A1		40.4 ± 12.8	40.4 ± 13.8	0.70
A2		42.3 ± 17.1	39.8 ± 16.4	0.11
A3		44.1 ± 16.6	39.2 ± 15.9	0.05
**Proteinuria (mg/g creatinine)**	All	194.5 (106.8-540.7)	205.6 (113.1-508.9)	0.94
A1		93.4 (79.3-122.8)	119.9 (102.4-175.7)	0.03^a^
A2		211.7 (158.7-277.9)	266.9 (112.1-339.9)	0.21
A3		1,285.6 (916.9-5,500.0)	1,199.1 (633.5-4,862.4)	0.14
**UACR (mg/g creatinine)**	All	49.3 (15.5-346.4)	62.3 (19.6-170.3)	0.23
A1		13.6 (10.1-18.1)	17.2 (9.7-41.3)	0.01^a^
A2		63.6 (48.8-94.8)	70.3 (24.9-125.5)	0.52
A3		937.2 (635.5-3,662.3)	813.9 (362.2-3,449.8)	0.09
**uα-1-MG (mg/g creatinine)**	All	29.6 (14.6-42.2)	35.5 (24.3-60.5)	<0.001^a^
A1		17.2 (11.5-30.6)	30.3 (22.8-42.7)	<0.001^a^
A2		35.1 (16.0-60.3)	38.8 (23.2-76.9)	0.006^a^
A3		34.6 (21.5-66.7)	36.4 (28.1-72.4)	0.30
**uDKK3 (pg/mg creatinine)**	All (n = 51)	546.5 (165.5-2,506.2)	745.5 (158.7-2,521.8)	0.87
A1 (n = 21)		456.5 (159.7-675.2)	745.5 (334.3–2,378.7)	0.36
A2 (n = 18)		559.2 (177.6-2,447.1)	300.0 (116.7-2,073.8)	0.71
A3 (n = 12)		2,522.0 (471.2-8,202.6)	1,616.9 (94.1-5,938.8)	0.27

*Note:* Unless otherwise specified, data are presented for the entire cohort (All, n = 57) and stratified by albuminuria stages: A1 (n = 22), A2 (n = 21), and A3 (n = 14). Baseline and month 6 values are reported as mean ± standard deviation for eGFR, and as median (interquartile range) for UACR, uα-1-MG, and uDKK3. *P*-values indicate results of paired statistical comparisons between baseline and month 6 within each group.

Abbreviations: uDKK3, urinary Dickkopf-3; eGFR, estimated glomerular filtration rate; UACR, urinary albumin creatinine ratio; uα-1-MG, urinary α-1-microglobulin.

a *P* < 0.05.

### Course of Proteinuria Within 6 Months of SGLT2I Therapy

Over all patients, there was no significant change in proteinuria within 6 months of SGLT2I therapy (*P* = 0.94; Table 2). Regarding the patients in respect to their different albuminuria stages, patients with stage A1albuminuria showed a significant increase in median proteinuria from 93.4 to 119.9 mg/g creatinine (*P* = 0.03), while stage A2 und A3 patients showed no significant change in median proteinuria (*P* = 0.21 and *P* = 0.14, respectively).

### Course of UACR Within 6 months of SGLT2I Therapy

**Figure 2:**
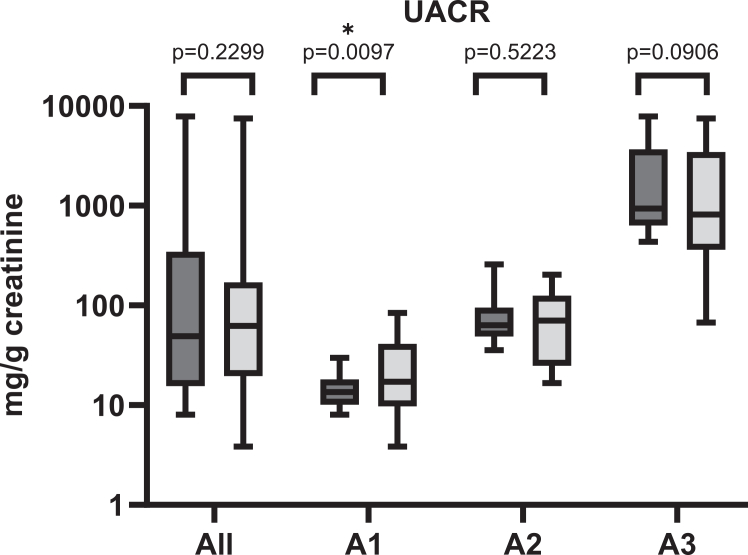
SchäferCourse of urinary albumin creatinine ratio under 6 months of SGLT2I therapy in patients with different urinary albumin creatinine ratio stages.

Over all patients, there was no significant change in the UACR within 6 months of SGLT2I therapy (*P* = 0.23; Table 2). Regarding the patients in respect to different albuminuria stages, patients with stage A1 showed a significant increase in median UACR from 13.6 to 17.2 mg/g creatinine (*P* = 0.01). Stage A2 patients showed no relevant change in median UACR (*P* = 0.52), and patients with A3 albuminuria showed a numerical reduction of median UACR from 937.2 to 813.9 mg/g creatinine, missing statistical significance (*P* = 0.09; Fig 2).

### Course of uα-1-MG Within 6 Months of SGLT2I Therapy

**Figure 3:**
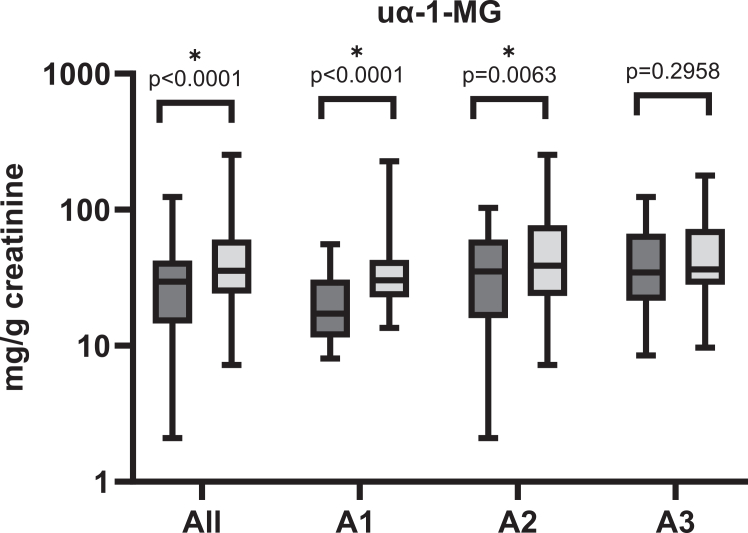
Course of urinary α1-microglobulin under 6 months of sodium-glucose cotransporter-2 inhibitors therapy in patients with different urinary albumin creatinine ratio stages.

After 6 months of SGLT2I therapy, there was a significant increase in median uα-1-MG from 29.6 to 35.5 mg/g creatinine in all patients (*P* < 0.001, Table 2). Differentiated according to the albuminuria groups, A1 and A2 patients showed a significant increase in median uα-1-MG from 17.2 to 30.3 mg/g creatinine (*P* < 0.001) and from 35.1 to 38.8 mg/g creatinine (*P* = 0.006), whereas in patients in stage A3 uα-1-MG was unchanged (*P* = 0.30; Fig 3). Adjusted for urinary creatinine, changes in uα-1-MG stayed significant in all and A1 patients (*P* = 0.007 and *P* = 0.005, respectively).

### Course of uDKK3 Within 6 Months of SGLT2I Therapy

**Figure 4:**
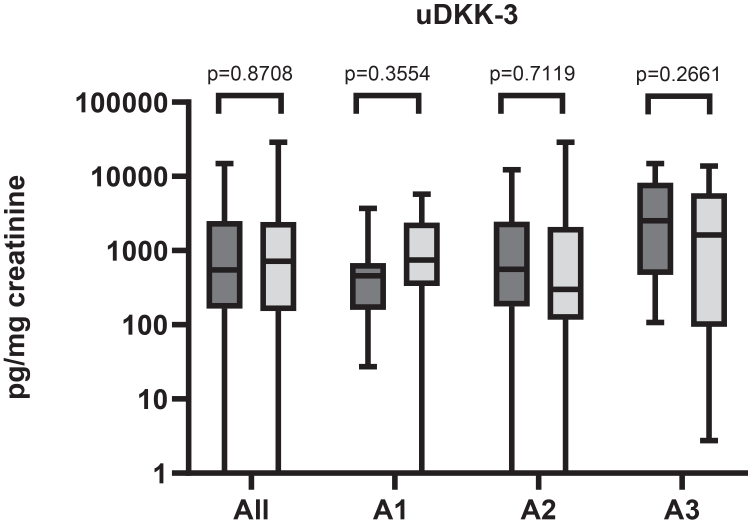
Course of urinary Dickkopf-3 under 6 months of sodium-glucose cotransporter-2 inhibitors therapy in patients with different urinary albumin creatinine ratio stages.

Regarding uDKK3 within 6 months of SGLT2I therapy, no significant changes of median uDKK3 levels were observed in the entire cohort, as well as in the different stages of albuminuria (*P* = 0.87 for all patients, *P* = 0.36 for A1 patients, *P* = 0.71 for A2 patients, and *P* = 0.27 for A3 patients, Fig 4 and Table 2).

### Correlation of the Change of uα-1-MG With Changes in eGFR and UACR

**Figure 5:**
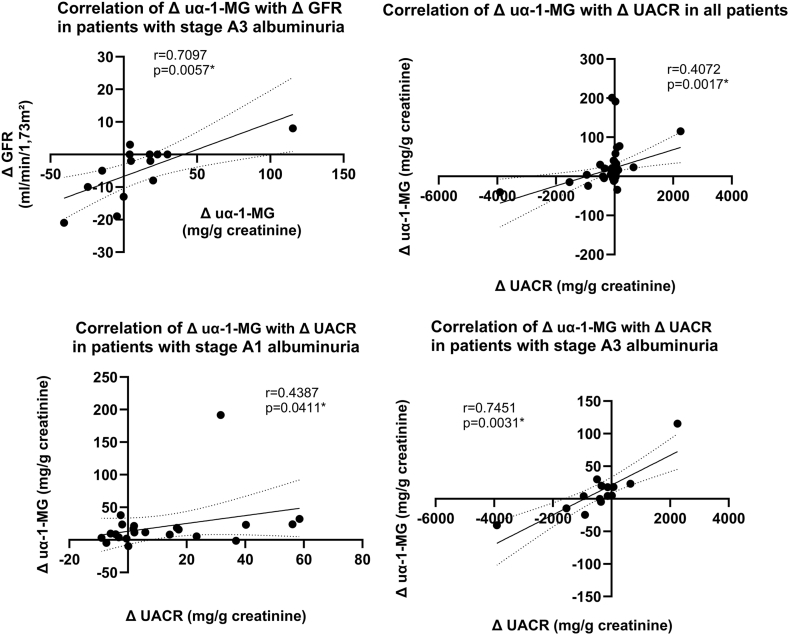
Correlation of the change of urinary α1-microglobulin with changes in estimated glomerular filtrartion rate and urinary albumin creatinine ratio.

No significant correlation was found between the change in uα-1-MG and the change in eGFR in all patients (*P* = 0.06), A1 patients (*P* = 0.37), and A2 patients (*P* = 0.78). A positive correlation between changes in uα-1-MG and eGFR was observed in stage A3 patients (r = 0.7097; *P* = 0.006) (Fig 5).

As for the correlation between changes in uα-1-MG and changes in UACR, a significant positive correlation was found in all patients (r = 0.4072; *P* = 0.002), stage A1 patients (r = 0.4387; *P* = 0.04), and stage A3 patients (r = 0.7451; *P* = 0.003) (Fig 5). No significant correlation was detected in A2 patients (*P* = 0.70).

### Analysis of Confounding Factors

Multiple linear regression analyses were performed to assess the influence of predefined covariates on changes in uDKK3, uα-1-MG, and UACR over 6 months of SGLT2I therapy. Changes in UACR were significantly associated only with baseline eGFR (estimate −0.00868; *P* = 0.0166). Age, sex, BMI, type 2 diabetes mellitus, HF status, and baseline UACR showed no significant association with UACR changes. For uα-1-MG, significant associations were observed for BMI (estimate −0.0208; *P* = 0.0082), type 2 diabetes mellitus (estimate 0.2226; *P* = 0.0063), and baseline uα-1-MG levels (estimate −0.00456; *P* = 0.0015). After adjustment for all covariates, the increase in uα-1-MG during SGLT2I therapy remained significant (estimate 0.3053; *P* = 0.0006). Age, sex, eGFR, and HF status were not significantly associated with changes in uα-1-MG. No covariate showed a significant association with changes in uDKK3 (Tables S1-S3).

## Discussion

SGLT2I therapy demonstrates outstanding cardiorenal benefits, although the underlying mechanisms are not yet fully understood. This study reveals a significant increase in uα-1-MG in patients with CKD treated with SGLT2I for 6 months, particularly pronounced in UACR stage A1. Notably, there were no changes in uDKK3, a marker of tubular epithelial stress and damage, while UACR remained stable during treatment.^19^ These findings highlight the differential impact of SGLT2I on glomerular and tubular function.

Historically, albuminuria in early diabetic nephropathy was attributed primarily to glomerular hyperfiltration or barrier dysfunction.^11^ However, increasing evidence suggests that proximal tubular dysfunction plays a significant role in the pathogenesis of albuminuria in diabetic and nondiabetic forms of kidney disease.^20^^,^^21^ Clinical studies in early nephropathies demonstrated a correlation between urinary albumin and tubular markers, suggesting early tubular involvement.^22^ In rat models, the presence of albuminuria in the absence of changes in the glomerular sieving coefficient suggests that the observed proteinuria is primarily attributable to impaired tubular reabsorption rather than increased glomerular permeability.^23^^,^^24^ This dysfunction has been linked to downregulated expression of the endocytic receptor megalin in proximal tubular cells.^23^ Evidence from preclinical studies supports a crosstalk between SGLT2I and megalin, both localized in the proximal tubules, which may underlie potential benefits for CKD.^4^ In this context, SGLT2I is expected to attenuate not only glucose overload but also protein overload in proximal tubular epithelial cells.^4^ In hyperfiltration states, excess tubular glucose reabsorption through SGLT2 increases O-linked β-N-acetylglucosamine modification of megalin, redistributing it to the epithelial surface, enhancing protein endocytosis, and leading to protein overload in the tubules followed by apoptosis, oxidative stress, and fibrosis.^4^^,^^25^, ^26^, ^27^, ^28^, ^29^ SGLT2I therapy may suppress this modification, promoting megalin internalization and reducing protein reabsorption, which might explain the observed increase in uα-1-MG, especially in A1 stage.^4^ Correspondingly, albuminuria and total proteinuria also increased or did not decrease in these stages where hyperfiltration plays a minor role. However, this remains a hypothetical mechanism and is not directly supported by the present data. Because SGLT2 is selectively expressed in the proximal tubules, inhibitors targeting this transporter may also influence the function of other tubular molecules^30^ and may impair proximal tubular reabsorption of highly filtered plasma proteins. In addition, osmotic stress may also play a role. By reducing sodium and glucose reabsorption in proximal tubules, SGLT2I increase solute delivery to downstream nephron segments, leading to osmotic diuresis and a hyperosmotic luminal environment. This may transiently impair protein reabsorption efficiency, contributing to an elevated uα-1-MG level.^31^^,^^32^

In this study, the relationship between uα-1-MG and changes in eGFR and UACR following SGLT2I treatment was assessed. No significant correlation between changes in uα-1-MG and eGFR was found in the overall cohort or the subgroup of patients in stages A1 and A2, suggesting that higher urinary uα-1-MG levels are not due to decreased filtration and subsequent serum accumulation. However, there was a significant positive correlation in patients with stage A3, suggesting that greater reductions in hyperfiltration, reflected by a larger eGFR decline during SGLT2I treatment in this group, may lower tubular protein load and alleviate tubular protein reabsoprtion, potentially counteracting the generally observed increased uα-1-MG excretion with SGLT2I therapy. The present results imply that at higher UACR stages (A3), SGLT2I effects are likely predominantly glomerular (eg, improved tubuloglomerular feedback), while at lower UACR stages (A1 and A2), tubular mechanisms are more influential. Correspondingly, albuminuria and total proteinuria numericaly decreased in A3 patients, whereas they increased in patients with A1 albuminuria and stayed stable in A2 patients with a numerical increase. The significant correlation between changes in uα-1-MG and UACR across the entire cohort and especially in stage A3 albuminuria, suggests that both α-1-MG and albumin excretion might be, at least partially, influenced by similar mechanisms and is also described previously for other tubular proteins.^22^ Additional evidence suggests that SGLT2I treatment is associated with increased tubular proteinuria.^4^ Inhibition of SGLT2 using antisense oligonucleotides elevated urinary β2-microglobulin and kidney injury molecule-1.^33^ Similar to the stage-dependant results of this study on uα-1-MG, in patients with diabetes with normoalbuminuria, SGLT2I therapy for 24 weeks increased urinary N-acetyl-β-D-glucosaminidase but decreased it in those with A3 albuminuria.^34^ Although luseogliflozin improved glomerular injury and renal fibrosis in diabetic rats, it was linked to increased proteinuria, likely due to reduced tubular reabsorption.^35^ Nevertheless, it remains unclear whether tubular proteinuria induced by SGLT2I treatment has different implications when compared with tubular proteinuria unrelated to SGLT2I treatment, which is typically associated with kidney damage and CKD progression,^4^^,^^36^ The increase in uα-1-MG during SGLT2I treatment may reflect nephroprotective modulation of protein handling and reduction of intracellular protein overload, a known contributor to tubulointerstitial injury. The absence of changes in uDKK3, a marker of tubular epithelial stress and damage,^16^^,^^37^ supports that the observed increase in uα-1-MG reflects a functional adaptation rather than structural tubular injury. Therefore, the increase in uα-1-MG observed during SGLT2I therapy offers a novel perspective on tubular protein handling in CKD. This mechanism could be part of the therapeutic modulation of tubular function that might contribute to the nephroprotective effects of SGLT2I. Given the complexity of interpreting tubular protein markers under SGLT2I treatment, further studies are needed to clarify these mechanisms and their implications for CKD management.

In this study, UACR remained unchanged over 6 months of SGLT2I treatment in the overall cohort. This may be due to opposing effects of reduced glomerular hyperfiltration lowering albumin filtration and impaired tubular albumin reabsorption. Consistent with the present data showing a numerical reduction in albuminuria only in stage A3, a meta-analysis of randomized trials in patients with diabetes found no effect of SGLT2I in those with normal to mildly (A1) or normal to mildly/moderate increased albuminuria (A1/A2), but a reduction in patients with moderate (A2) or severe (A3) albuminuria.^38^ Notably, in this study UACR increased in A1 patients, underscoring the role of proximal tubular dysfunction in the development of albuminuria. The potential inverse influence of the eGFR on the development of UACR corresponds to the frequent clinical observation of an association of higher UACR stages with lower filtration rates.

This study has several limitations, including its monocentric character, its sample size and its observational nature, which may restrict the generalizability of the findings. Because there is no control group, it is unclear whether the changes in biomarkers observed correspond to normal changes in patients with CKD over time or reflect an effect of SGLT2I therapy of unknown magnitude.

In the multiple-adjusted model, BMI, the presence of type 2 diabetes mellitus, and baseline uα-1-MG levels were associated with changes in uα-1-MG during SGLT2I therapy.

The inverse association between BMI and uα-1-MG changes may reflect obesity-associated glomerular hyperfiltration, which is mitigated by SGLT2I and may consequently reduce tubular protein load. In contrast, the presence of type 2 diabetes mellitus was associated with a greater increase in uα-1-MG, suggesting a distinct pathophysiological mechanism in diabetic patients.

Furthermore, higher baseline uα-1-MG levels were associated with smaller subsequent increases, possibly indicating that underlying tubular mechanisms were already activated before initiation of SGLT2I therapy. Of importance, after adjustment for all identified confounders, the increase in uα-1-MG during SGLT2I therapy remained highly significant, supporting a direct effect of SGLT2 inhibition on tubular protein handling that appears to be independent of these factors. Nevertheless, given the multifactorial nature of CKD and the complex interplay between glomerular and tubular mechanisms, the clinical significance of increased uα-1-MG requires further exploration. Although this study supports the potential of uα-1-MG as a marker of SGLT2I effect on tubular protein handling, its utility as a standalone marker of tubular damage may be limited by its UACR stage-dependent behavior in CKD. Additionally, direct evidence linking SGLT2I-induced modulation of megalin to uα-1-MG increases was not assessed. Incorporating molecular and histological evaluations in future research may provide deeper insights into these mechanisms. Moreover, the duration of follow-up was limited to 6 months, which may not capture the very early and the long-term effects of SGLT2I on tubular proteinuria.

In conclusion, this study shows that SGLT2I therapy leads to increased uα-1-MG excretion in patients with CKD, particularly in patients with lower hyperfiltration, which manifests itself in a lower UACR stage. Because uDKK3 remained stable as a parameter for tubular stress and damage, these results suggest that the increase in uα-1-MG reflects a functional adaptation of the proximal tubule that reduces tubular protein overload rather than indicating tubular damage. The results of this study underscore the differential effects of SGLT2I on tubular and glomerular function, with stage-dependent effects on tubular proteinuria depending on whether the glomerular or tubular effect predominates. Further studies are needed to clarify whether increased tubular proteinuria under SGLT2I therapy contributes to the nephroprotective effects of SGLT2I through the associated reduction in tubular protein overload.

## References

[1] Heerspink H.J.L., Stefánsson B.V., Correa-Rotter R. (2020). Dapagliflozin in patients with chronic kidney disease. N Engl J Med.

[2] The EMPA-KIDNEY Collaborative Group, Herrington W.G., Staplin N. (2023). Empagliflozin in patients with chronic kidney disease. N Engl J Med.

[3] Cherney D.Z.I., Perkins B.A., Soleymanlou N. (2014). Renal hemodynamic effect of sodium-glucose cotransporter 2 inhibition in patients with type 1 diabetes mellitus. Circulation.

[4] Otomo H., Nara M., Kato S. (2020). Sodium-glucose cotransporter 2 inhibition attenuates protein overload in renal proximal tubule via suppression of megalin O-GlcNacylation in progressive diabetic nephropathy. Metabolism.

[5] Iordan L., Gaita L., Timar R., Avram V., Sturza A., Timar B. (2024). The renoprotective mechanisms of sodium-glucose Cotransporter-2 inhibitors (SGLT2i)-A narrative review. Int J Mol Sci.

[6] Upadhyay A., SGLT (2024). Inhibitors and kidney protection: mechanisms beyond tubuloglomerular feedback. Kidney360.

[7] Gekle M. (1998). Renal proximal tubular albumin reabsorption: daily prevention of albuminuria. News Physiol Sci.

[8] Tojo A., Endou H. (1992). Intrarenal handling of proteins in rats using fractional micropuncture technique. Am J Physiol.

[9] Teppo A.-M., Honkanen E., Finne P., Törnroth T., Grönhagen-Riska C. (2004). Increased urinary excretion of alpha1-microglobulin at 6 months after transplantation is associated with urinary excretion of transforming growth factor-beta1 and indicates poor long-term renal outcome. Transplantation.

[10] Thielemans N., Lauwerys R., Bernard A. (1994). Competition between albumin and low-molecular-weight proteins for renal tubular uptake in experimental nephropathies. Nephron.

[11] Zeni L., Norden A.G.W., Cancarini G., Unwin R.J. (2017). A more tubulocentric view of diabetic kidney disease. J Nephrol.

[12] Grubb A. (1992). Diagnostic value of analysis of cystatin C and protein HC in biological fluids. Clin Nephrol.

[13] Christensen E.I., Birn H. (2002). Megalin and cubilin: multifunctional endocytic receptors. Nat Rev Mol Cell Biol.

[14] Verroust P.J., Birn H., Nielsen R., Kozyraki R., Christensen E.I. (2002). The tandem endocytic receptors megalin and cubilin are important proteins in renal pathology. Kidney Int.

[15] Penders J., Delanghe J.R. (2004). Alpha 1-microglobulin: clinical laboratory aspects and applications. Clin Chim Acta.

[16] Schunk S.J., Speer T., Petrakis I., Fliser D. (2021). Dickkopf 3-a novel biomarker of the “kidney injury continuum”. Nephrol Dial Transplant.

[17] Schunk S.J., Zarbock A., Meersch M. (2019). Association between urinary dickkopf-3, acute kidney injury, and subsequent loss of kidney function in patients undergoing cardiac surgery: an observational cohort study. Lancet.

[18] Zewinger S., Rauen T., Rudnicki M. (2018). Dickkopf-3 (DKK3) in urine identifies patients with short-term risk of eGFR loss. J Am Soc Nephrol.

[19] Federico G., Meister M., Mathow D. (2016). Tubular Dickkopf-3 promotes the development of renal atrophy and fibrosis. JCI Insight.

[20] Christensen E.I., Gburek J. (2004). Protein reabsorption in renal proximal tubule-function and dysfunction in kidney pathophysiology. Pediatr Nephrol.

[21] Amsellem S., Gburek J., Hamard G. (2010). Cubilin is essential for albumin reabsorption in the renal proximal tubule. J Am Soc Nephrol.

[22] Gibb D.M., Tomlinson P.A., Dalton N.R., Turner C., Shah V., Barratt T.M. (1989). Renal tubular proteinuria and microalbuminuria in diabetic patients. Arch Dis Child.

[23] Tojo A., Onozato M.L., Ha H. (2001). Reduced albumin reabsorption in the proximal tubule of early-stage diabetic rats. Histochem Cell Biol.

[24] Russo L.M., Sandoval R.M., Campos S.B., Molitoris B.A., Comper W.D., Brown D. (2009). Impaired tubular uptake explains albuminuria in early diabetic nephropathy. J Am Soc Nephrol.

[25] Zhuang Y., Yasinta M., Hu C. (2015). Mitochondrial dysfunction confers albumin-induced NLRP3 inflammasome activation and renal tubular injury. Am J Physiol Ren Physiol.

[26] Nishi Y., Satoh M., Nagasu H. (2013). Selective estrogen receptor modulation attenuates proteinuria-induced renal tubular damage by modulating mitochondrial oxidative status. Kidney Int.

[27] Fang L., Xie D., Wu X., Cao H., Su W., Yang J. (2013). Involvement of endoplasmic reticulum stress in albuminuria induced inflammasome activation in renal proximal tubular cells. PLOS One.

[28] Takagaki Y., Shi S., Katoh M., Kitada M., Kanasaki K., Koya D. (2019). Dipeptidyl peptidase-4 plays a pathogenic role in BSA-induced kidney injury in diabetic mice. Sci Rep.

[29] van Timmeren M.M., Bakker S.J.L., Vaidya V.S. (2006). Tubular kidney injury molecule-1 in protein-overload nephropathy. Am J Physiol Ren Physiol.

[30] Iida T., Hosojima M., Kabasawa H. (2022). Urinary A- and C-megalin predict progression of diabetic kidney disease: an exploratory retrospective cohort study. J Diabetes Complications.

[31] Marton A., Saffari S.E., Rauh M. (2024). Water conservation overrides osmotic diuresis during SGLT2 inhibition in patients with heart failure. J Am Coll Cardiol.

[32] Dickenmann M., Oettl T., Mihatsch M.J. (2008). Osmotic nephrosis: acute kidney injury with accumulation of proximal tubular lysosomes due to administration of exogenous solutes. Am J Kidney Dis.

[33] van Meer L., van Dongen M., Moerland M., de Kam M., Cohen A., Burggraaf J. (2017). Novel SGLT2 inhibitor: first-in-man studies of antisense compound is associated with unexpected renal effects. Pharmacol Res Perspect.

[34] Nunoi K., Sato Y., Kaku K., Yoshida A., Suganami H. (2018). Effects of sodium-glucose cotransporter 2 inhibitor, tofogliflozin, on the indices of renal tubular function in patients with type 2 diabetes. Endocrinol Diabetes Metab.

[35] Kojima N., Williams J.M., Takahashi T., Miyata N., Roman R.J. (2013). Effects of a new SGLT2 inhibitor, luseogliflozin, on diabetic nephropathy in T2DN rats. J Pharmacol Exp Ther.

[36] Makhammajanov Z., Gaipov A., Myngbay A., Bukasov R., Aljofan M., Kanbay M. (2024). Tubular toxicity of proteinuria and the progression of chronic kidney disease. Nephrol Dial Transplant.

[37] Dziamałek-Macioszczyk P., Winiarska A., Pawłowska A., Wojtacha P., Stompór T. (2023). Patterns of Dickkopf-3 serum and urine levels at different stages of chronic kidney disease. J Clin Med.

[38] Piperidou A., Sarafidis P., Boutou A. (2019). The effect of SGLT-2 inhibitors on albuminuria and proteinuria in diabetes mellitus: a systematic review and meta-analysis of randomized controlled trials. J Hypertens.

