# Supplementary material for: Effects of SGLT2I Therapy on Tubular Reabsorption and Tubular Epithelial Stress Injury in Patients With CKD
Source: Kidney Med. 2026 May 26;8(8):101416. doi: 10.1016/j.xkme.2026.101416 (PMC13312476; doi:10.1016/j.xkme.2026.101416)
Supplement: Supplementary File (PDF) — Tables S1-S3 [file mmc1.pdf]

Supplementary Material

Multiple linear regression			
**Dependent variable**	Delta UACR		
**Regression type**	Least squares		
**Parameter estimates**	**Variable**	**Estimate**	**p-value**
β0	Intercept	0.09060	0.4290
β1	Age	-0.004985	0.3346
β2	Gender	-0.1090	0.2978
β3	Type II Diabetes mellitus	-0.02720	0.8207
β4	Heart Failure	-0.03300	0.7691
β5	eGFR	-0.008683	0.0166*
β6	BMI	0.001667	0.8841
β7	Basline UACR	-4.277e-005	0.2354
**R squared**	0.1525		

Table S1 Multiple linear regression to analyze the development of UACR during 6 months of SGLT2I therapy using the least squares model. To meet the requirement of normal distribution, the change in UACR was calculated from the baseline values logarithmized on a base of 10 and the values logarithmized on the same basis after 6 months of SGLT2I therapy. The confounding factors analyzed were age, gender, BMI, eGFR, type II diabetes mellitus, heart failure status, and baseline UACR. The parameters age, BMI, and eGFR were centered on the mean value of the total cohort**.** BMI – body mass index, eGFR - estimated glomerular filtration rate, SGLT2I - Sodium-glucose cotransporter-2 inhibitor, UACR – urinary albumin-to-creatinine ratio.

Multiple linear regression			
**Dependent variable**	Delta uα-1-MG		
**Regression type**	Least squares		
**Parameter estimates**	**Variable**	**Estimate**	**p-value**
β0	Intercept	0.3053	0.0006*
β1	Age	-0.002095	0.4943
β2	Gender	-0.02634	0.7007
β3	Type II Diabetes mellitus	0.2226	0.0063*
β4	Heart Failure	-0.07910	0.2865
β5	eGFR	0.0001252	0.9571
β6	BMI	-0.02083	0.0082*
β7	Basline uα-1-MG	-0.004556	0.0015*
**R squared**	0.2758		

Table S2 Multiple linear regression to analyze the development of u*α*-1-MG during 6 months of SGLT2I therapy using the least squares model. To meet the requirement of normal distribution, the change in uα-1-MG was calculated from the baseline values logarithmized on a base of 10 and the values logarithmized on the same basis after 6 months of SGLT2I therapy. The confounding factors analyzed were age, gender, BMI, eGFR, type II diabetes mellitus, heart failure status, and baseline uα-1-MG. The

parameters age, BMI, and eGFR were centered on the mean value of the total cohort. BMI – body mass index, eGFR - estimated glomerular filtration rate, SGLT2I - Sodium-glucose cotransporter-2 inhibitor, uα-1-MG – urinary alpha-1 microglobulin.

**Multiple linear regression**			
**Dependent variable**	Delta uDKK3		
**Regression type**	Least squares		
**Parameter estimates**	**Variable**	**Estimate**	**p-value**
β0	Intercept	0.09188	0.7429
β1	Age	-0.009606	0.4101
β2	Gender	0.1821	0.4871
β3	Type II Diabetes mellitus	-0.02240	0.9437
β4	Heart Failure	-0.06974	0.8134
β5	eGFR	-0.002314	0.8037
β6	BMI	-0.02783	0.3362
β7	Basline uDKK3	-6.707e-005	0.1074
**R squared**	0.1073		

Table S3 Multiple linear regression to analyze the development of uDKK3 during 6 months of SGLT2I therapy using the least squares model. To meet the requirement of normal distribution, the change in uDKK3 was calculated from the baseline values logarithmized on a base of 10 and the values logarithmized on the same basis after 6 months of SGLT2I therapy. The confounding factors analyzed were age, gender, BMI, eGFR, type II diabetes mellitus, heart failure status, and baseline uDKK3. The parameters age, BMI, and eGFR were centered on the mean value of the total cohort. BMI – body mass index, eGFR - estimated glomerular filtration rate, SGLT2I - Sodium-glucose cotransporter-2 inhibitor, uDKK3 – urinary Dickkopf-3.
